# Rapid Prototyping in Orthopaedic Surgery: A User's Guide

**DOI:** 10.1100/2012/838575

**Published:** 2012-05-01

**Authors:** Mark Frame, James S. Huntley

**Affiliations:** Orthopaedic Department, Royal Hospital for Sick Children, Yorkhill, Glasgow G3 8SJ, UK

## Abstract

Rapid prototyping (RP) is applicable to orthopaedic problems involving three dimensions, particularly fractures, deformities, and reconstruction. In the past, RP has been hampered by cost and difficulties accessing the appropriate expertise. Here we outline the history of rapid prototyping and furthermore a process using open-source software to produce a high fidelity physical model from CT data. This greatly mitigates the expense associated with the technique, allowing surgeons to produce precise models for preoperative planning and procedure rehearsal. We describe the method with an illustrative case.

## 1. Background

Rapid prototyping is a manufacturing technology used in many industries to develop high fidelity three-dimensional structures from source image data. Medical applications have generally been within an academic centre or research environment, as support costs (expertise, software, and equipment) have been large. Applications within clinical practice include preoperative planning/conceptualisation, procedure rehearsal [1–7], and educational tools for teaching [8] and patient communication [9].

The idea of using computed tomography (CT) data to build physical models was put forward by Alberti in 1979 [10]. In 1979, a polystyrene model of a pelvis was constructed so that a custom-made metal implant could be designed for a patient with fibrosarcoma [11]. In the 1980s there was considerable progress in model building from CT data using polystyrene and then the stronger polyurethane foam [12]. Milled models were quite accurate for larger structures, for example, being subject to average deviation of only 1.6 mm for distances between high-resolution structures [12, 13]. By 1985, 3D imaging had progressed to a level at which image fidelity was sufficient for more widespread clinical use at academic centres [14, 15] to plan for complex surgery, especially using computed tomography (CT) of the craniofacial and maxillofacial regions [6, 13, 16, 17]. In 1994, Zonneveld and Fukuta highlighted the difficulty in data conversion from that of standard “slice-oriented” segmented object files to formats used in model manufacture [15].

There are several RP techniques [1, 2]. Milling is a *subtractive* technology, in which a foam block is successively trimmed away to produce the model. The *additive* techniques include stereolithography, selective laser sintering [18], the solider process, fused deposition moulding, laminated object manufacturing, and 3D printing.

Using computer software to produce 3D reconstructions and to calculate volumes has been useful in many specialties in medicine including hepatobiliary surgery [19, 20]. These reports comment on the difficulties with this technique, particularly access to software. Most of the software was inaccessible to the nonradiologist, being bundled with the hardware used to produce the scans. Some free or non-CT-scanner-based software has been developed to try an overcome this problem [21, 22], for example, MacMeasure on the Apple Macintosh [23] and ImageJ, a free software package developed by the National Institute of Health (NIH) [19, 24, 25].

The majority of literature regarding RP in surgery focuses on its uses within the maxillofacial specialty [13, 16, 17, 26]. There is a limited but growing body of work concerning innovative uses within orthopaedics and trauma surgery, for example, for preoperative planning [27], particularly in spinal surgery where placement of screws requires extreme accuracy [28]. Custom fabrication of orthopaedic jigs using RP techniques has now become a commercially viable option for arthroplasty of the knee as introduced by Biomet with the Signature knee system in collaboration with Materialise. Further developments include the fabrication of complete custom implants [29] and bioscaffolds for bone tissue engineering [30–35].

## 2. Illustrative Case

A 12-year-old boy sustained fractures of both right (dominant) forearm diaphyses by falling on his outstretched hand whilst playing football. He underwent surgery in the form of open reduction of his ulna and single-bone intramedullary wiring. The wire and cast were both removed at four weeks, and he was advised to start moving the arm. Although substantial deformity was noted, a course of observation was selected.

**Figure 1:**
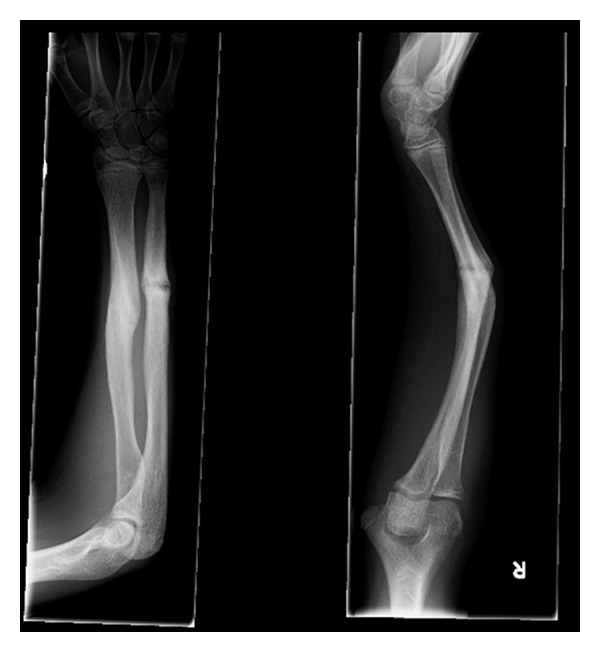
The original deformity—orthogonal views right forearm.

Eleven months after his index procedure, he was referred to our tertiary unit with an established malunion. On examination, he had obvious deformity (apex dorsal ~40°), no focal tenderness, intact neurovasculature, and pronation/supination limited to 40°/0° from neutral. Radiographs (Figure 1) show the deformity.

**Figure 2:**
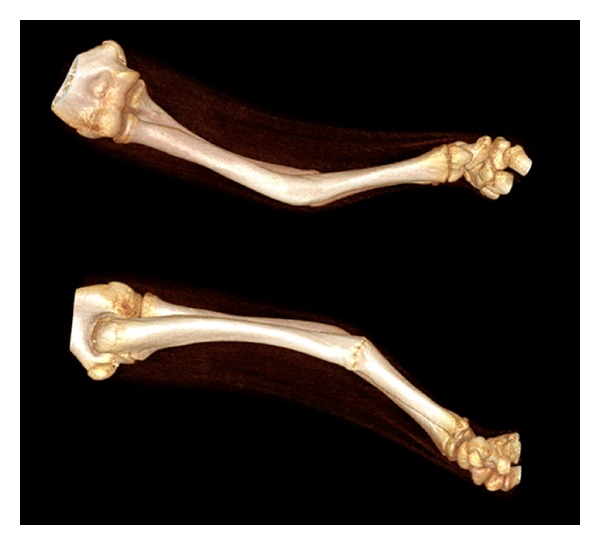
3D reconstructions of CT data showing the right forearm deformity.

Computed tomography with 3D reconstruction was performed (Figure 2).

Blood tests (including full blood count, C-reactive protein, and erythrocyte sedimentation rate) were all within reference ranges.

Three-dimensional osteotomies for malunited fractures of the upper limb are not performed commonly [36, 37] and are potentially awkward not merely because of the angular deformities and remodelling [38] but also because of the possible effects of rotational malunion of both bones [39]. Computer simulation has been found useful elsewhere [40, 41]. Assessment of the 3D-recontructed images suggested that the rotational component to the deformities of the radius and ulna was minimal, that is, the distal and proximal landmarks of each bone appeared to have maintained their usual rotational relations.

**Figure 3:**
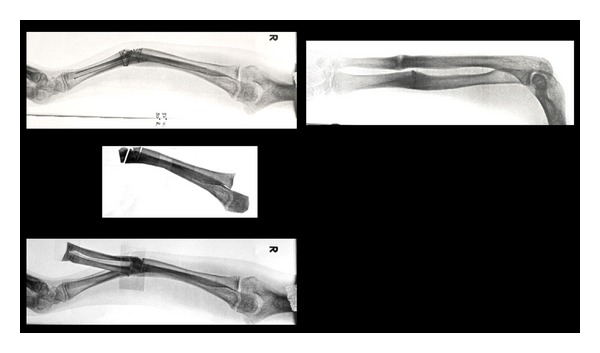
Preoperative planning—defining the osteotomies.

Preoperative planning (Figure 3) used paper, scissors and tape and would have been facilitated by 3D prototypes of the bones involved.

The operation involved two closing wedge osteotomies (uniplanar for the ulna and biplanar for the radius). The excised radial wedge was used as bone graft at the ulna and direct compression plates (3.5 mm) were used in compression mode. Nonsteroidal anti-inflammatory drugs were excluded postoperatively.

**Figure 4:**
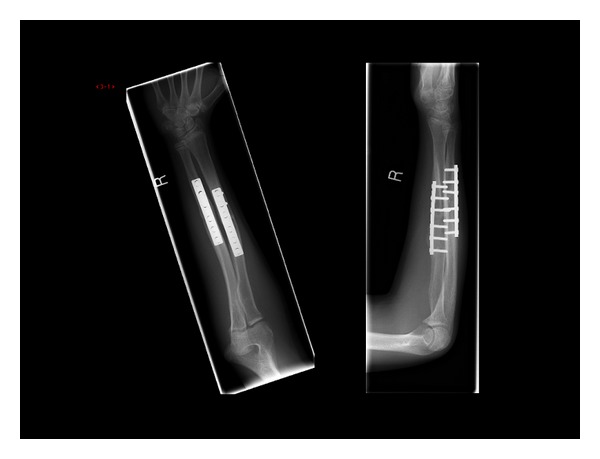
Postoperative results—orthogonal views at twelve weeks.

A cast was used post-operatively for 4 weeks, and radiographs were obtained at 6 weeks and 12 weeks (Figure 4). At 12 weeks, he had clinical and radiographic union, and pronation/supination had improved to 75°/90°.

Whilst dealing with this case of malunion of the shaft of radius and ulna, CT scanning with 3D formatting was performed to aid preoperative planning. These images suggested that there was no rotational component to the malunion and that accordingly the osteotomies could be planned relatively easily in a conventional manner. However, as an adjunct it was hoped to use the DICOM data to build a model of the malunited bones. A university research department was involved, and several problems were encountered: (i) time delay due to file conversion problems, (ii) only a truncated model was produced, and (iii) the angle of malunion did not conform to that obtained on CT scan, that is, the model was not an accurate representation.

The cost of RP is primarily determined by the amount of the material used, the cost of the 3D printer, and the cost of the software licenses required to process the DICOM data to a suitable format to print. With this in mind, our aim was to find an appropriate method to produce an acceptable orthopaedic RP model (i) at minimal cost, (ii) without access to a university research department, and (iii) using open-source software and a public-access 3D printing service. A secondary aim was to compare the cost involved against quotations from established companies.

## 3. Method

Before models were produced or the patient's images processed, informed consent was obtained for use of case material and CT images/data for research and publication.

The CT scans of the patient's forearm were then processed using open-source software OsiriX (DICOM image processing software for OS X) and MeshLab (a system for the processing and editing of unstructured 3D triangular meshes). Both packages are distributed under open-source licensing—Lesser General Public Licence (LGPL)—and are therefore free. The resultant files were uploaded and printed using Shapeways.com, a commercial company providing public access to 3D printing.

## 4. From DICOM to Model

### 4.1. CT Scanning

Standard DICOM data from the patients CT scan were transferred to CDROM. This was imported into OsiriX.

### 4.2. OsiriX Processing

**Figure 5:**
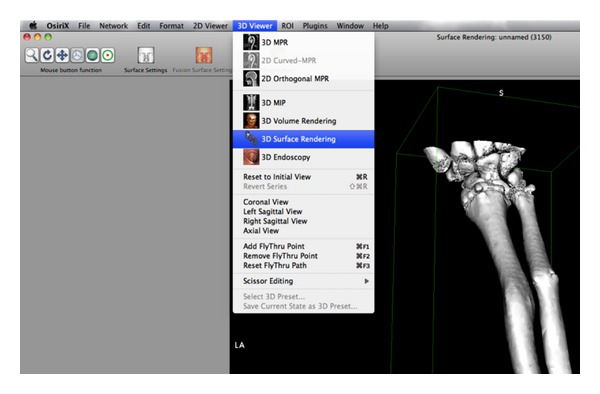
Surface rendering.

Once in OsiriX, the series was “double-clicked” to open it into the standard 2D viewing windows. From the “3D Viewer” drop down menu, “3D Surface Render” was selected and the standard defined values were accepted (Figure 5).

**Figure 6:**
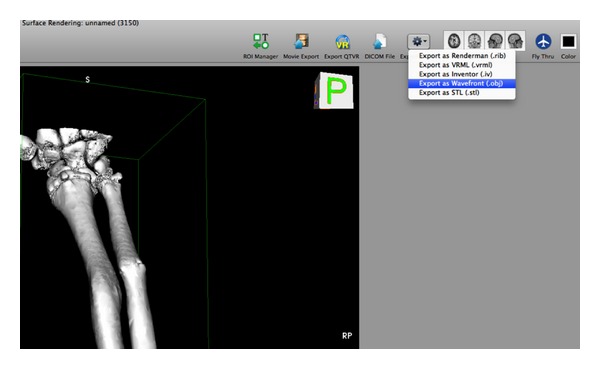
Exporting as an  .obj file.

 A surface render was produced which converted the images into a 3D data point “mesh” that can be exported from OsiriX as an  .obj file (Figure 6). An  .obj file is a geometry definition format originally developed by Wavefront Technologies. The file format is open and has been adopted by other 3D graphics applications.

### 4.3. Mesh Processing—MeshLab

**Figure 7:**
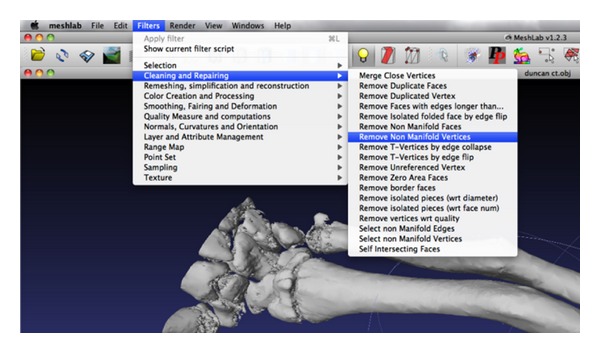
“Cleaning” of the mesh.

This  .obj file was then opened within MeshLab (http://meshlab.sourceforge.net/). MeshLab is an open-source system for the processing and editing of unstructured 3D triangular meshes. This software allows the manipulation of the 3D image to remove unwanted artefact and to isolate specific bones or sections. The mesh must be “cleaned” by removing duplicated, unreferenced vertices, null face and using automatic filling of holes if required (Figure 7). This is done from a drop down menu under “filters.” Once these operations have been performed, the mesh is saved as an  .stl file.

STL is a file format native to the stereolithography computer-aided design (CAD) software created by 3D Systems (Rock Hill, SC). This format is supported by many software packages and is widely used for rapid prototyping and computer-aided manufacturing. Importantly this file contains only data points and does not contain patient-identifying information. This file can be uploaded to a commercial 3D printer for production.

### 4.4. Cost Comparisons

Several commercial companies offer an RP service. Cost comparisons were made from quotations. Seven companies were identified in response to the Google query <3D printing> (accessed 09/07/10). A further company (Materialise http://www.materialise.com/) was added because of reports [3, 7, 42] of its ability to prototype for orthopaedic applications. Companies were approached for a quote to build a 3D rapid prototype of both forearm bones from CT-DICOM data for a patient with a malunion after fracture (it was indicated that standard DICOM data could be supplied). Seven companies replied—all requiring file conversion to an appropriate format. Quote estimates for printing of converted files were supplied by 3 companies— *£*420 ± 40 (mean ± SEM; *n* = 3). Quotes for file conversion from DICOM data were given by 2 companies ((i) *£*480, (ii) *£*85/hr). Therefore an estimate of the expense of acquiring a 3D rapid prototype of both forearm bones from CT-DICOM data, using commercially available avenues, is *£*500–900.

### 4.5. 3D Printing

Having performed the file conversions/processing ourselves, we used a company based in The Netherlands: Shapeways.com. They do not specialise in any specific industry but provide public access to 3D printing. They provide a fast service (less than 10-day turnaround) and charge based on volume in cm^3^ and the material used to produce the model. We used a plastic-like material PA 2200, which is printed using the technique of Selective-Laser-Sintering (SLS). SLS uses a high power laser (e.g., a carbon dioxide laser) to fuse small particles of plastic or metal, ceramic, or glass powders into the designated object. PA 2200 is a white nylon, which is strong and flexible. It is also one of their most detailed (minimum detail 0.2 mm) materials. Minimum wall thickness of structures printed can be 0.7 mm and maximum size of printed structure can be 31 × 23 × 18 cm (http://www.shapeways.com/materials/white_strong_flexible—accessed 19/09/11). It is stable up to a temperature of 80°C.

### 4.6. Assessment of the Model

**Figure 8:**
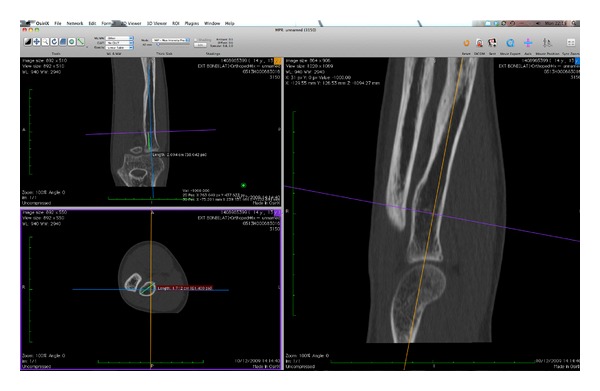
Measurements on CT scan.

The models produced were then validated against the 3D CT scan images using digital electronic vernier calipers to take multiple measurements at defined intervals (20 mm) along the model bones and at the same points on the CT scan (Figure 8). Two observers measured the same segments independently on three separate occasions.

The measurements were analysed for a statistically significant difference with a paired Student's *t*-test. The *P* values showed no statistical difference in dimensions between the printed models and the original CT scans (see Tables 1 and 2).

### 4.7. Model Production

**Figure 9:**
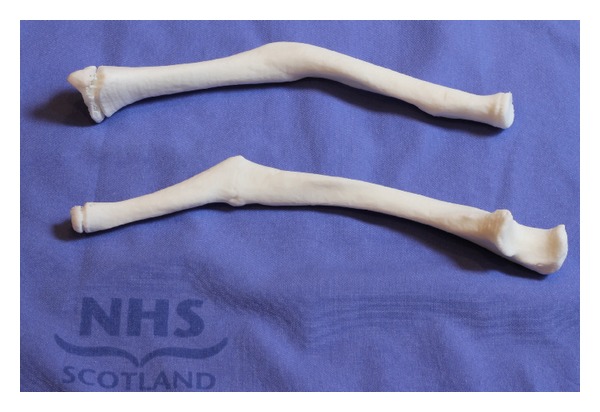
“Model” radius and ulna.

**Figure 10:**
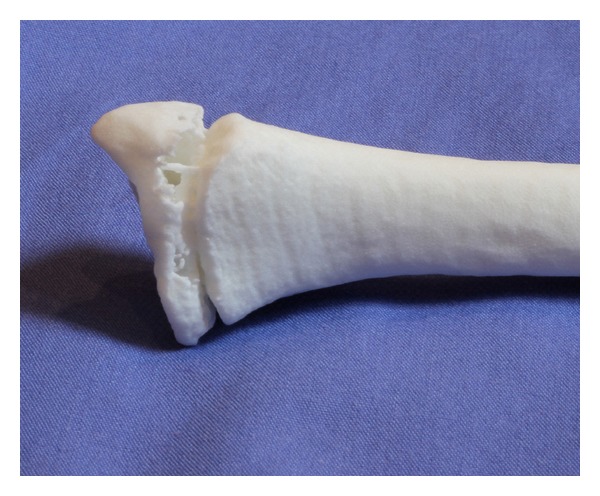
Distal radius showing level of physeal detail.

We produced a 3D reproduction of malunited forearm bones from standard CT DICOM data using free open-source software. The models cost a total of *£*77 including shipping to the department for both the radius and ulna (Figures 9 and 10) and took only 7 days to arrive.

## 5. Discussion

The main objective was to establish a simple and cost-effective method to produce accurate and detailed physical 3 dimensional models from standard CT scans. We aimed to do this quickly without specialist expertise. With the use of OsiriX and Meshlab on a standard personal computer and a publicly accessed commercial online 3D printing service, an inexpensive (*£*77 compared with *£*500–*£*900) model suitable for preoperative planning can be produced in a short time frame (7 days). The data from the measurements confirmed the fidelity of the model produced. Other reports have used the technique of SLS [18]. However, none are known to have used Osirix [22] to manipulate the data for this purpose.

The measurements and validation of 3D models can undoubtedly be improved and are the subject of further work. This study focused on simple-shaped long bones with thick periosteum allowing for noncomplex image processing. The process can be used for more complex shapes and problems for example, complex fracture patterns and structures with extensive soft tissue coverage such as the adult pelvis or hip joint.

Because of the reduction in financial outlay, this valuable technology is now in the reach of most orthopaedic departments allowing for models for teaching, preoperative planning, and operation rehearsal (we have used this method to construct adult hip models for pathological situations including cam impingement, slipped femoral epiphysis, and developmental dysplasia). There need be no reliance on links with university/research departments.

## Figures and Tables

**Table 1:** 

Radius	3D Model	CT Model	% Difference
Styloid	25.52	26.1	2.23
	16.18	16.8	3.69
	15.10	14.78	−2.14
	19.44	19.67	1.16
	16.20	16.8	3.57
	14.76	14.64	−0.79
	13.18	13.8	4.51
	14.36	14.2	−1.10
	16.94	17.12	1.04
Head	19.35	18.2	−6.29

*P* = 0.1383.

**Table 2:** 

Ulna	3D Model	CT Model	% Difference
Styloid	11.16	11.5	2.97
	11.44	11.76	2.72
	14.55	14.43	−0.85
	23.38	23.21	−0.73
	16.78	16.3	−2.94
	14.44	14.9	3.07
	13.39	13.17	−1.67
	14.65	14.87	1.51
	18.26	18.16	−0.54
Head	23.87	23.51	−1.51

*P* = 0.8283.
